# A unique case report of bilateral spontaneous cerebrospinal fluid leak from bilateral sphenoid sinus skull base defects

**DOI:** 10.4102/jcmsa.v4i1.271

**Published:** 2026-03-20

**Authors:** Divya P. Ramyead Basant Rai, Lien Deschuytere, Darlene Lubbe

**Affiliations:** 1 Division of Otorhinolaryngology (ENT), Department of Surgery, Faculty of Health Sciences, University of Cape Town, Cape Town, South Africa

**Keywords:** spontaneous cerebrospinal fluid leak, Sternberg’s canal, idiopathic intracranial hypertension, transpterygoid, lateral sphenoid sinus recess

## Abstract

This brief report describes the presentation and management of a rare case of bilateral spontaneous cerebrospinal fluid (CSF) leaks resulting from skull base dehiscence in the sphenoid sinus lateral recess. A retrospective review of clinical data, including imaging and surgical interventions, was conducted. Informed consent was obtained. A middle-aged, obese woman presented with clear rhinorrhoea. Imaging identified bilateral encephaloceles and skull base defects. She underwent successful endoscopic surgery with fat grafts and septal flap repair, with no CSF leak recurrence at follow-ups.

**Contribution:**

To our knowledge, this case is one of the few documented examples of bilateral spontaneous CSF leaks from skull base dehiscence in the sphenoid sinus. Endoscopic sinus surgery provides a minimally invasive, effective repair. Anatomical variations, such as extensive sphenoid sinus pneumatisation and congenital defects, combined with factors such as high body mass index, may predispose patients to spontaneous CSF leaks.

## Introduction

This case report describes a clinical scenario involving bilateral spontaneous cerebrospinal fluid (CSF) leaks resulting from skull base dehiscence in the sphenoid sinus recesses. Encephaloceles within the lateral recess of the sphenoid sinus are rare entities that should be considered in patients presenting with spontaneous CSF rhinorrhoea. The objective of this report is to have a closer look at the pathophysiology behind this rare case.

## Methods

A retrospective review of the electronic medical record was used to collect information pertaining to the patient’s clinical history and management.

## Case report

A 54-year-old woman, with no previous history of head trauma and meningitis, presented with a 2-month history of spontaneous clear fluid leakage from both nostrils, exacerbated by bending forward. During the medical history interview, the patient reported numbness over both cheeks and headaches which were alleviated by lying down. On examination, her weight was recorded at 123 kg, her height at 1.67 m, and her body mass index (BMI) was calculated at 44.1. Nasal examination revealed evidence of previous sinus surgery and a posterior septal perforation.

**FIGURE 1:**
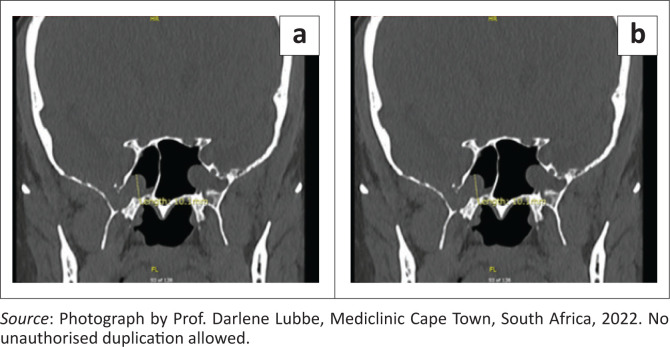
Coronal view of non-contrasted computed tomography scan of paranasal sinus showing bilateral sphenoid sinus encephaloceles.

A non-contrasted computed tomography (CT) scan of the paranasal sinuses revealed an encephalocele measuring approximately 10 mm in diameter located in the inferolateral aspect of the right sphenoid sinus, along with a defect in the lateral wall of the right sphenoid sinus, lateral to the foramen rotundum (FR), measuring 5.2 mm × 5.2 mm. Another meningoencephalocele was noted in the lateral aspect of the left sphenoid sinus, measuring 1.7 cm × 1.3 cm × 1.4 cm, also lateral to the FR (Figure 1).

**FIGURE 2:**
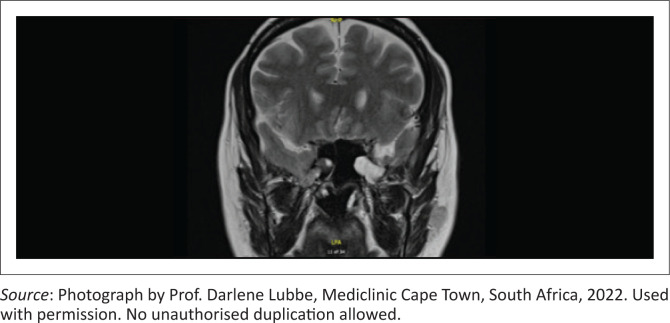
Magnetic resonance imaging scan of paranasal sinus showing isointense encephaloceles in bilateral sphenoid sinuses.

Subsequently, a magnetic resonance imaging (MRI) scan was performed, revealing a right sphenoid encephalocele and a left meningoencephalocele extending through a defect located lateral to the FR (Figure 2). The diffusion-weighted imaging (DWI) sequence was normal, with no extra-axial collections or intracranial mass lesions. Intracranial flow voids appeared normal, and post-contrast imaging showed no signs of pathologic intracranial enhancement.

Intraoperatively, bilateral meningoencephaloceles were identified lateral to the vidian canal and FR. A right-sided full-length mucoperichondrial septal flap was raised from a hemitransfixion incision. Residual left mucoperichondrial septal mucosa was left intact to allow later rotation over the vomerine edge. A complete bilateral sphenoidectomy was performed. Using a contralateral nostril, transpterygoid approach was used. To reach the most lateral recess of the left sphenoid sinus, the left sphenopalatine artery as well as the vidian artery and nerve were sacrificed. On the right, via endonasal approach, the entire anterior face of the sphenoid sinus was drilled back to the level of the vidian canal to fully expose the defect. Bilateral encephaloceles were reduced using bipolar cautery until the bony margins of the defect were clearly visualised. Using a 30-degree endoscope, the defects were packed with abdominal fat and DuraGen™ (Integra LifeSciences Corporation, Plainsboro Township, New Jersey) to ensure a watertight closure. Reconstruction was completed using the large nasoseptal flap pedicled on the right sphenopalatine artery, draped across the posterior sphenoid wall to also cover the contralateral defect. Duraseal^®^ (Confluent Surgical Inc., Waltham, MA, US) secured the repair. The left middle turbinate was sutured to the right to support the reconstruction.

**FIGURE 3:**
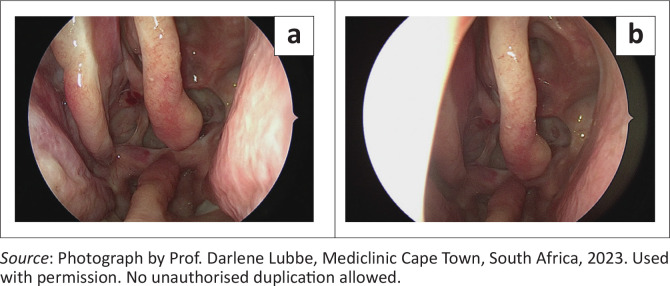
Follow-up endoscopic view 1 year later. Posterior septectomy and bilateral septal flaps visualised. No cerebrospinal fluid leak.

The patient was post-operatively placed on prophylactic acetazolamide for 3 months and sodium valproate for 2 weeks. Follow-up appointments were conducted at 2 weeks, 6 weeks, and 3 months. Follow-up endoscopic view 1 year later showed no cerebrospinal leak (Figure 3). A telephonic conversation with the patient after 3 years revealed no recurrence of either CSF leak during this period.

## Discussion

The sphenoid sinus can be variable in size, shape and pneumatisation. Its complete development is only achieved at age of 20 years. However, at 6–7 years, secondary pneumatisation occurs leading to the lateral wall of the sinus (formed by a line joining the vidian canal and FR) to extend beyond the pterygoid process, greater wing of sphenoid, foramen ovale, middle cranial fossa and lateral to the cavernous sinus.^1^ In 1888, Maximilian Sternberg described a congenital lateral craniopharyngeal canal, also known as Sternberg’s canal, which occurs from incomplete fusion of the greater sphenoid wing with the basisphenoid.^2^ A myriad of papers have been published in literature with regard to its location with respect to FR and vidian canal and CSF leak classification systems evolved. Chandrakiran et al.^3^ reported seven patients with spontaneous CSF leaks lateral to the FR, while Tababee et al.^4^ documented 13 patients with defects medial to V2. In 2020, Hanz et al.^5^ proposed a classification system with four types of CSF leaks: Type I, Sternberg canal; type II, medial to FR; type III, lateral to FR; and type IV, both medial and lateral with FR enlargement. Tosi et al.^6^ used these proposed four types of CSF leaks on a multi-institutional cohort of patients with lateral sphenoid encephaloceles to determine their incidence and any correlation with surgical outcome. Until recently in 2025, Manogaran et al.^7^ proposed a comprehensive algorithm for managing surgical interventions in sphenoid lateral recess CSF leaks based on radiological classifications and anatomic parameters.

The pathophysiology behind spontaneous CSF leaks could vary from congenital skull base defects to acquired causes. Lateral recess pneumatisation, attenuated roof of the sphenoid sinus recess and skull base thinning can all lead to spontaneous CSF leaks. Underlying idiopathic intracranial hypertension (IIH) is often associated with spontaneous CSF leaks and leads to arachnoid pits, encephalocele formation and empty sella syndrome. Cerebrospinal fluid drainage from the arachnoid granulation to the dural venous sinus may be impaired when CSF pressure increases, leading to progressive enlargement and scalloping of the underlying bone.^8,9^ A female predominance has been found across literature. Kumar et al.^10^ found that women with IIH have significantly higher CSF testosterone levels, which increases the CSF output in the choroid plexus. Furthermore, women in the childbearing age group had a higher prevalence because of higher circulating sex hormones. Obesity is also a contributory factor as adipose tissue releases inflammatory cytokines, which in turn lead to the secretion of mineralocorticoids.^11^

Spontaneous CSF leaks caused by multiple skull base defects are extremely rare, with only a few cases documented in the literature. Ramsden et al.^12^ documented the first case of bilateral spontaneous CSF rhinorrhoea from the cribriform plate. Nadeem et al.^13^ described one case of a concomitant defect involving both the sphenoid and ethmoid sinuses. Dallan et al.^14^ found that, of 25 patients with multiple CSF leaks, 21 (84%) had two simultaneous defects, 3 (12%) had three simultaneous defects, and 1 (4%) had four simultaneous defects. Most of the concurrent skull base defects were situated in the tegmen tympani, cribriform plate, ethmoidal roof, posterior table frontal sinus, olfactory fissure. To the best of our knowledge, this is the first reported case of bilateral spontaneous CSF leaks from the lateral wall of both sphenoid sinuses.

Endoscopic sinus surgery has revolutionised the management of spontaneous sphenoid CSF leaks owing to its minimally invasive nature and enhanced visualisation capabilities. Endoscopic transpterygoid approach, transmaxillary approach, transsphenoidal approach, contralateral precaruncular approach and lateral transorbital approach are alternative approaches for repairing CSF leaks.^15^ A multiportal surgical approach allows enhanced visualisation of the anatomical site and multisurgeon access, with multiple surgical instruments. In our case, the bilateral defects were successfully repaired during a single surgery, using the endoscopic transpterygoid approach on the left and a direct endonasal approach on the right. This approach allowed direct exposure of both defects.

A successful repair is further supported by lowering intracranial pressure (ICP) through weight reduction with healthy nutrition and post-operative acetazolamide. Lumbar drains are not routinely used but may be considered when far lateral defects present technical difficulty. The senior author uses a protocol of draining 10 mL of CSF from the lumbar drain every 2 h for 48 h when a lumbar drain is inserted. This prevents complications that could occur from accidental overdraining and allows for patients to be managed in a ward setting post-operatively. Postoperative care includes 48 h of bed rest and strict avoidance of activities that raise ICP, such as nose blowing, straining, or lifting heavy objects; prophylactic stool softeners are routinely recommended.^16^

Idiopathic intracranial hypertension is characterised by increased ICP of unknown cause, producing papilloedema, headache and visual loss.^17^ Limitations in our case report include the lack of routinely testing patients for IIH post-operatively through fundoscopy or lumbar puncture. Patients only receive a post-operative brain CT if they complain of headaches to rule out hydrocephalus. Only patients that present with further CSF rhinorrhoea are extensively investigated for IIH to decide on the need for possible ventriculoperitoneal (VP) shunting.

## Conclusion

To conclude, bilateral spontaneous CSF leaks from the sphenoid sinuses are rare. This case highlights the successful management of bilateral spontaneous CSF leaks originating from the lateral walls of the sphenoid sinuses using endoscopic techniques. Endoscopic surgery with various approaches can be used, but the senior author feels that this case could have benefitted from a bilateral lateral transorbital approach instead.
